# Sending Nudes: Sex, Self-Rated Mate Value, and Trait Machiavellianism Predict Sending Unsolicited Explicit Images

**DOI:** 10.3389/fpsyg.2017.02210

**Published:** 2017-12-18

**Authors:** Evita March, Danielle L. Wagstaff

**Affiliations:** School of Health Sciences and Psychology, Federation University Australia, Ballarat, VIC, Australia

**Keywords:** explicit images, short-term mating, online dating, dark personality, mate value

## Abstract

Modern dating platforms have given rise to new dating and sexual behaviors. In the current study, we examine predictors of sending unsolicited explicit images, a particularly underexplored online sexual behavior. The aim of the current study was to explore the utility of dark personality traits (i.e., narcissism, Machiavellianism, psychopathy, and sadism) and self-rated mate value in predicting attitudes toward and behavior of sending unsolicited explicit images. Two hundred and forty participants (72% female; *M*age = 25.96, *SD* = 9.79) completed an online questionnaire which included a measure of self-rated mate value, a measure of dark personality traits, and questions regarding sending unsolicited explicit images (operationalized as the explicit image scale). Men, compared to women, were found to have higher explicit image scale scores, and both self-rated mate value and trait Machiavellianism were positive predictors of explicit image scale scores. Interestingly, there were no significant interactions between sex and these variables. Further, Machiavellianism mediated all relationships between other dark traits and explicit image scale scores, indicating this behavior is best explained by the personality trait associated with behavioral strategies. In sum, these results provide support for the premise that sending unsolicited explicit images may be a tactic of a short-term mating strategy; however, future research should further explore this claim.

## Introduction

The surge of increased access to the Internet has brought along changes in mate selection, and the Web is now one of the most popular places to find a date or a romantic partner (Fitzpatrick et al., 2015). Although online dating offers significant benefits (see Finkel et al., 2012; Clemens et al., 2015), this modern platform has generated new forms of dating, and subsequently, sexual behavior. ‘Sexting’ is defined as sending sexually suggestive messages, either using explicit language or nude/nearly nude photos and videos (Delevi and Weisskirch, 2013) and has previously been referred to as a high risk behavior, especially among youths (Döring, 2014). Recent prevalence rates show that 10.2% of adolescents (Klettke et al., 2014) and 30–54% of adults (Döring, 2014) have previously sent a sext containing sexually suggestive text or photo content. Despite the apparently prolific nature of this behavior, empirical research exploring individual difference predictors of sending nudes remains significantly bereft. The current study will explore one particular online dating behavior that has received very limited attention in the literature, specifically, the sending of pictures of one’s own genitals (i.e., explicit images).

Sending sexts which contain pictures of one’s genitals can be categorized in two ways: solicited (when one asks to receive such images) or unsolicited (when one receives an image one has not asked for). Receiving unsolicited images of genitals is increasingly common when online dating (Ley, 2016). In fact, when online dating, women’s receipt of an unsolicited picture of men’s genitals (known colloquially as ‘dick pics’; Salter, 2015) is often the first communication many report receiving (Ley, 2016).

Although previous research has explored the occurrence of sexting in a committed romantic relationship (e.g., Drouin and Landgraff, 2012), little research has explored sending unsolicited explicit images to strangers. Of this, Tziallas (2015) explored these behaviors on the male homosexual location-based real-time dating applications (or apps), Grinder and Scruff. Participants reported primarily positive reactions at receiving an unsolicited dick pic. These results are in direct contrast to reports of women who indicate that unsolicited dick pics are unwanted, can be shaming, and may even be a form of online sexual harassment (Vitis and Gilmour, 2016).

In addition to the limited literature on sending unsolicited explicit images, individual differences for engaging in this behavior are not well-understood. Previous research has shown people may send sexts to seek reciprocal behavior, express pride with one’s body (Salter, 2015), to initiate sexual activity (Henderson and Morgan, 2011), and even as a deviant sexual behavior (Ahern and Mechling, 2013; Klettke et al., 2014). In the current research, we explore predictors of sending unsolicited explicit images in an attempt to establish if this behavior is better predicted by deviant personality traits (i.e., dark personality traits), or by mate quality. Specifically, we explore if this a sexually deviant behavior, or is this an advertisement of mate quality.

Primarily, sexting has been labeled a sexually deviant behavior (Döring, 2014), as regardless of whether the participants are consenting adults, the behavior is generally not considered socially acceptable (Reyns et al., 2014). Furthering the deviance of this behavior, sexting has been associated with risky behaviors such as alcohol and drug consumption (Benotsch et al., 2013), and unprotected sex (Davis et al., 2016). Considering the proposed deviance of this behavior, we expect personality traits associated with perpetration of deviant behaviors will predict sending unsolicited explicit images. The personality traits of narcissism, Machiavellianism, psychopathy, and sadism (the Dark Tetrad; see Chabrol et al., 2009) are all considered socially aversive (Paulhus and Williams, 2002) and are associated with engagement in antisocial behavior (Jones and Paulhus, 2010). These dark personality traits have been associated with more deviant sexual tendencies, including coercion (Figueredo et al., 2015), more positive attitudes toward rape (Jonason et al., 2017), and repeated sexual advances (Zeigler-Hill et al., 2016). In particular, trait psychopathy and sadism have been strongly associated with perpetration of sexually deviant behavior (Williams et al., 2009; Buckels et al., 2013). The positive association between dark traits of personality and sexually exploitative behavior could extend to sending unsolicited explicit images, which have previously been defined as a form of online sexual harassment (Vitis and Gilmour, 2016). Thus, if sending unsolicited explicit images is a sexually deviant behavior, then we expect that individuals with higher levels of dark personality traits (particularly psychopathy and sadism) will be more inclined to engage in this behavior.

Alternatively, engaging in this potentially risky sexual behavior may not only be due to deviant personality traits. Rather, such risky sexual behavior might be a sexual strategy, as previous research has shown both men and women prefer short-term mates who are risk takers over risk avoiders (Sylwester and Pawłowski, 2011). Given the risks associated with sending sexual images (particularly unsolicited ones) including reputational damage (Hudson et al., 2014), sending explicit images may act as a signal of one’s willingness to engage in risky behaviors, therefore acting as a signal of their mate value. Further, individuals who engage in sending unsolicited explicit images may adopt a ‘nothing gained nothing lost’ strategy toward sexual encounters, where the cost of a missed opportunity outweighs the risk of rejection (Joel et al., 2017). When adopting such a strategy, it is reasonable to suggest those individuals high in mate value would benefit the most, as they would be least likely to be rejected.

Finally, it is also possible that the sending of unsolicited explicit images is associated with a false-positive bias when interpreting sexual intent (e.g., Haselton, 2003). Such false-positive bias would lead individuals to send unsolicited explicit images, as they may genuinely believe the person they are sending it to will enjoy receiving it, and this would be particularly relevant for individuals with higher self-rated mate value. Further, as higher self-rated mate value in men is associated with higher sexual over-perception (Kohl and Robertson, 2014), this may go some way to explaining why women indicate that an unsolicited dick pic is unwanted and perceived as a form of online sexual harassment, compared to the positive reactions of men (Vitis and Gilmour, 2016). Thus, if sending unsolicited explicit images is a mate quality advertisement^1^, then self-rated mate value should be associated with the sending of explicit images, and this should be more prominent in men than women.

The aim of the current study was to establish the utility of dark personality traits (i.e., narcissism, Machiavellianism, psychopathy, and sadism) and self-rated mate value in predicting behavior and attitude toward sending unsolicited explicit images. We predicted that all dark personality traits and mate value would positively, significantly predict these attitudes and behavior. Further, we predicted that men, compared to women, would have a more positive attitude toward and engage in higher perpetration of sending unsolicited explicit images.

## Materials and Methods

### Participants and Procedure

Two hundred and forty participants^2^(72% female; *M*age = 25.96, *SD* = 9.79) were recruited online via social media and snowballing. All participants met the criteria for inclusion in the current study, which was responding ‘YES’ to having previously sent an unsolicited explicit image (i.e., an image of their own genitals that the other person did not explicitly ask for). The majority of participants indicated that they were heterosexual (70.4%), followed by bisexual (18.3%), homosexual (6.3%), or ‘other’ (5.0%). Participants accessed the survey online via advertising material, and after consenting to participate completed each of the scales in random order. This study formed part of a larger survey, and total participation time was approximately 30 min. This study was carried out in accordance with the recommendations of the Federation University Human Research Ethics Committee with informed consent from all subjects. All subjects gave informed consent in accordance with the Declaration of Helsinki. The project was approved by the Federation University Human Research Ethics Committee (Project Number: 16-167A).

### Materials

Materials for the current study included an online questionnaire with a number of measures. Participant’s self-rated mate value was assessed with the Mate Value Scale (Edlund and Sagarin, 2014), a four-item scale where participants respond to statements such as “Overall, how do you believe you compare to other people in desirability as a partner on the following scale?” on a seven-point Likert scale (1 = *Very much lower than average*; 7 = *Very much higher than average*). Scores were summed to form an overall index of mate value, with the current study demonstrating high internal reliability (α = 0.88).

Individual narcissism was measured using the NPI-16 (Ames et al., 2006). Participants responded Yes (2) or No (1) to 16 statements (e.g., *I really like to be the center of attention*). Responses were summed to form an overall index of narcissism, with high internal reliability (α = 0.82).

Psychopathy was measured using the Levenson’s Psychopathy Scale (Levenson et al., 1995), a 26-item scale where participants respond to statements such as “For me, what’s right is whatever I can get away with” on a five-point Likert scale (1 = *Strongly disagree*; 5 = *Strongly agree*). Scores were summed for a total score of psychopathy, and the scale showed high internal consistency (α = 0.84).

Individual Machiavellianism was measured with the MACH-IV (Christie and Geis, 1970), a 20-item scale where participants respond to statements such as “Never tell anyone the real reason you did something unless it is useful to do so” on a five-point Likert scale (1 = *Strongly disagree*; 5 = *Strongly agree*). Scores were summed for a total score of Machiavellianism, and the scale showed reasonable internal consistency (α = 0.70).

Sadism was measured using the Short Sadistic Impulse Scale (O’Meara et al., 2011), a 10-item scale where participants respond to statements such as “I enjoy seeing people hurt” on a five-point Likert scale (1 = *Strongly disagree*; 5 = *Strongly agree*). Scores were summed for a total score of sadism, and the scale showed high internal consistency (α = 0.87).

Finally, participant’s attitudes and behaviors regarding sending unsolicited explicit images were assessed with three questions constructed by the researchers. Participants were asked to respond to the following two statements on a five-point Likert scale (1 = *Strongly disagree*; 5 = *Strongly agree*): “I think others enjoy receiving pictures of my own genitals,” and “I enjoy sending explicit pictures of my own genitals to other people.” Finally, participants indicated the number of people they had sent unsolicited explicit images to on a five-point Likert scale (0 = *None^3^*, 1 = *One or two people*, 2 = *A few people*, 3 = *Four to ten people*, 4 = *More than 10 people*). Participant responses to these items were summed, and for the purpose of the study termed the explicit image scale (α = 0.80).

## Results

Descriptive statistics for each scale are shown in **Table 1**.

**Table 1:** Total descriptive values and sex differences for mate value, narcissism, Machiavellianism, psychopathy, sadism, and explicit image scale.

	Total		Men		Women				
						
	*M*	*SD*	*M*	*SD*	*M*	*SD*	*D*	*t*	*d*
Mate Value Scale	20.41	4.28	20.09	3.93	20.48	4.43	-0.39	-0.63	0.09
Narcissism	5.08	3.84	6.25	3.94	4.55	3.67	1.70	3.09^∗∗^	0.45
Machiavellianism	52.40	9.55	54.24	9.36	51.42	9.28	2.82	2.09^∗^	0.30
Psychopathy	59.18	14.84	62.23	15.88	57.81	13.99	4.42	2.07^∗^	0.30
Sadism	17.55	7.90	20.00	8.83	16.57	7.23	3.43	3.07^∗∗^	0.43
EIS	7.71	3.41	8.69	3.40	7.22	3.19	1.47	3.10^∗∗^	0.45


*EIS, explicit image scale; D, sex difference; ^∗∗^*p* < 0.01, ^∗^*p* < 0.05; values derived from corrected independent-samples *t*-tests (two-tailed); *d*, Cohen’s *d.**

**Table 1** shows men were significantly higher than women on narcissism, Machiavellianism, psychopathy, sadism, and explicit image scale. To assess appropriate inclusion of predictor variables in a regression model, bivariate correlations between predictor variables and criterion were assessed (see **Table 2**).

**Table 2:** Pearson’s correlations between predictors of sex, mate value, narcissism, Machiavellianism, psychopathy, and sadism, and criterion of explicit image scale.

	1	2	3	4	5	6
(1) Sex						
(2) Mate Value Scale	0.04					
(3) Narcissism	-0.20^∗∗^	0.26^∗∗∗^				
(4) Machiavellianism	-0.14^∗^	-0.14^∗^	0.23^∗∗∗^			
(5) Psychopathy	-0.12	-0.06	0.26^∗∗∗^	0.68^∗∗∗^		
(6) Sadism	-0.20^∗∗^	0.03	0.27^∗∗∗^	0.54^∗∗∗^	0.67^∗∗∗^	
(7) EIS	-0.20^∗∗^	0.16^∗∗^	0.23^∗∗∗^	0.26^∗∗∗^	0.19^∗∗∗^	0.22^∗∗∗^


*Sex coded as 1, men; 2, women; EIS, explicit image scale; multiple correlations corrected; ^∗∗∗^*p* < 0.001; ^∗∗^*p* < 0.01; ^∗^*p* < 0.05; multicollinearity not present (*r* < 0.7).*

The criterion of explicit image scale correlated positively with mate value, narcissism, Machiavellianism, psychopathy, and sadism, and negatively with sex, supporting their inclusion in a regression model. A multiple linear regression was conducted with sex, mate value, narcissism, Machiavellianism, psychopathy, and sadism as the predictors and explicit image scale as the criterion. The total model was significant [*F*(6,211) = 5.03, *p* < 0.001], explaining 12.5% (adjusted R^2^) of the variance in explicit image scale scores. Coefficients and partial correlations can be seen in **Table 3**.

**Table 3:** Coefficients for sex, mate value, narcissism, Machiavellianism, psychopathy, and sadism predicting explicit image scale.

Variables	*B*	*SE*	β	*t*	*pr*
Constant	2.29	2.15			
Sex	-1.02	0.49	-0.14	-2.08^∗^	-0.14^∗^
Mate Value Scale	0.12	0.05	0.16	2.37^∗^	0.16^∗^
Narcissism	0.05	0.06	0.06	0.82	0.06
Machiavellianism	0.07	0.03	0.19	2.15^∗^	0.15^∗^
Psychopathy	-0.02	0.02	-0.08	-0.82	-0.06
Sadism	0.06	0.04	0.13	1.49	0.10


*^∗^*p* < 0.05; *pr*, partial correlation.*

In an effort to explore possible moderation of sex and the significant predictors of mate value and Machiavellianism, interactions were calculated between sex and centralized mate value and Machiavellianism variables. When added to the model, these interaction variables did not explain any additional variance (*p* = 0.256).

**FIGURE 1:**
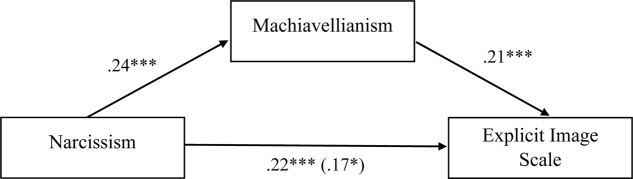
Standardized regression coefficients for the relationship between narcissism and explicit image scale as mediated by Machiavellianism. Standardized regression coefficient for the relationship between narcissism and explicit image scale when mediator is present is in parentheses; ^∗∗∗^*p* < 0.001; ^∗∗^*p* < 0.01; ^∗^*p* < 0.05.

**FIGURE 2:**
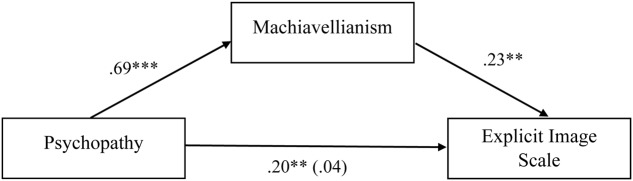
Standardized regression coefficients for the relationship between psychopathy and explicit image scale as mediated by Machiavellianism. Standardized regression coefficient for the relationship between psychopathy and explicit image scale when mediator is present is in parentheses; ^∗∗∗^*p* < 0.001; ^∗∗^*p* < 0.01; ^∗^*p* < 0.05.

**FIGURE 3:**
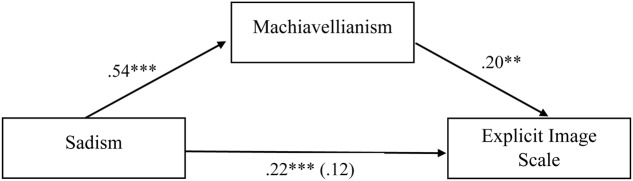
Standardized regression coefficients for the relationship between sadism and explicit image scale as mediated by Machiavellianism. Standardized regression coefficient for the relationship between sadism and explicit image scale when mediator is present is in parentheses; ^∗∗∗^*p* < 0.001; ^∗∗^*p* < 0.01; ^∗^*p* < 0.05.

Of interest was the significant bivariate correlations between narcissism, psychopathy, and sadism and the explicit image scale, but the non-significant partial correlations. Considering the conceptual overlap between all four dark personality traits (Furnham et al., 2013), we explored whether Machiavellianism was mediating the relationship between each of these variables and the explicit image scale. Using Hayes (2013) PROCESS Macro, three mediation models were run (see **Figures 1**–**3**).

**Figures 1**–**3** indicate that Machiavellianism partially mediated the relationship between narcissism and the explicit image scale, and fully mediated the relationship between psychopathy and the explicit image scale, and sadism and the explicit image scale.

## Discussion

The aim of the current study was to establish the utility of sex, self-rated mate value, and dark personality traits in predicting attitudes toward and perpetration of sending unsolicited explicit images of one’s genitals to others. Results showed utility for sex, mate value, and trait Machiavellianism in predicting this attitude and behavior. Men, compared to women, had higher explicit image scale scores, individuals with higher self-rated mate value had higher explicit image scale scores, and individuals with higher trait Machiavellianism had higher explicit image scale scores. There were no interactions between self-rated mate value and sex, and Machiavellianism and sex. Thus, although men may have more positive attitudes toward and perpetrate more sending of unsolicited explicit images, this does not moderate the utility of self-rated mate value and Machiavellianism to predict explicit image scale scores.

The utility of the dark personality traits predicting positive attitudes toward and perpetration of sending unsolicited explicit images offer an interesting insight into the potential ‘deviant’ nature of this behavior. As these dark traits have previously been associated with other sexually deviant behaviors, such as sexual coercion (Figueredo et al., 2015), positive rape attitudes (Jonason et al., 2017), and repeated unwanted sexual advances (Zeigler-Hill et al., 2016), the lack of utility in narcissism, psychopathy, and sadism in predicting this behavior was surprising; particularly as trait psychopathy and sadism are strong predictors of sexual deviancy (Williams et al., 2009; Buckels et al., 2013). As previous research has not yet explored dark personality traits and the sending of unsolicited explicit images, interpretation of these results are speculative. However, based on the utility of Machiavellianism to independently predict attitudes toward and perpetration of sending unsolicited explicit images, and the mediating nature of Machiavellianism and all other dark personality traits, it is possible that these explicit images are an ‘aggressive’ mating strategy, rather than a manifestation of deviant personality traits. More so than the other dark personality traits, individuals with high levels of Machiavellianism are apt behavioral strategists, effectively (and charmingly) exploiting situations and others for their own benefit (Book et al., 2015; Jonason et al., 2015). Thus, unlike the need for admiration associated with trait narcissism (e.g., Book et al., 2015), the callous nature associated with trait psychopathy (e.g., Lilienfeld et al., 2014), and the enjoyment of inflicting suffering associated with trait sadism (Buckels et al., 2013), it appears the trait best predictive sending unsolicited explicit images is that associated with strategic manipulation of others and situations.

The significance of self-rated mate value as a predictor also highlights the potential for sending unsolicited explicit images to be a mating strategy. Specifically, given *both* Machiavellianism and self-rated mate value were significant predictors, the sending of explicit images could be a more aggressive tactic for manipulating another individual into a short-term sexual interaction. Rather than simply signaling one’s mate quality, such an aggressive tactic could be particularly effective for individuals who are high in mate value. A limitation of this research was that short-term mating orientation and sexual tactics were not directly measured, and so the utility of these variables in predicting the sending of explicit images remains to be seen.

Interestingly, self-rated mate value remained a significant predictor of explicit image scale scores even when considering gender in the model. While men were still higher on the explicit image scale than women, it is possible that women’s online dating behavior differs from men’s less than what is observed in face-to-face relationship interactions. This is supported by evidence demonstrating that women act in an aggressive manner by trolling others on online dating apps as much as men do (March et al., 2017). The differential utility of sending unsolicited explicit images as it relates to mate value between men and women should therefore continue to be explored.

### Limitations and Future Directions

The nature of the current study was exploratory; thus, although results are informative they are still descriptive in nature and therefore interpretation is speculative. Further, a number of limitations are important to address. A significant limitation of the current study is the self-report nature of the questions relating to the sending of explicit images. In an effort to appear socially desirable, it is possible that men may have overestimated and women underestimated their engagement in and enjoyment of this behavior; still, considering the behavior in question an objective measure may prove hard.

A further limitation is the unexplained variance in the explicit image scale. A range of other factors are also likely to predict the sending of explicit images; for example, recipient reactions when receiving these images. It is reasonable to assume a range of individual differences exist between those who continue to send explicit images after receiving a negative or positive reaction from the recipient. Considering the propensity individuals with higher levels of these dark traits have for sexually exploiting others (e.g., Jonason and Webster, 2012), future research could explore whether recipient reactions moderate engaging in this behavior. Such results could further establish if engaging in this behavior is a mechanism to facilitate short-term mating (in which case correlations with offline sexual behavior should be observed), or is more akin to a sexually deviant behavior.

In summary, behavior and attitudes toward the sending of unsolicited explicit images is associated with being male, higher self-rated mate-value, and Machiavellianism, all of which suggest the sending of explicit images could be an extreme form of short-term mating strategy. While speculative, this study is the first to explore what motivations individuals might have to engage in this behavior online, and thus opens up new avenues for investigation.

## Author Contributions

EM and DW: Helped construct idea for research, collected data, ran analyses, helped produce drafts.

## Conflict of Interest Statement

The authors declare that the research was conducted in the absence of any commercial or financial relationships that could be construed as a potential conflict of interest.

## Footnotes

1 Thank you to an anonymous reviewer for suggesting this term for the behavior.

2 241 participants completed the questionnaire, with 1 participant not meeting inclusion criteria.

3 The category of ‘None’ was included as a further screening tool for exclusion of participants who had not previously sent an unsolicited explicit image. One participant selected this category, and therefore were excluded from analyses (*N* = 240).
